# Urethral Catheter Drainage Treatment of Intraperitoneal Bladder Injury Following Cesarean Section: A Case Report, Clinical Approach, and Brief Review of Literature

**DOI:** 10.7759/cureus.75216

**Published:** 2024-12-06

**Authors:** Moath K Alfentoukh, Faisal A Alrawaf, Nasser Almohaya, Mohammad Mahzari, Sami M Abuanz, Abdelrahman Y Mohammed, Rami M Hasan

**Affiliations:** 1 Urology, King Fahad Military Medical Complex, Dhahran, SAU

**Keywords:** cesarean section, conservative therapy, iatrogenic bladder injury, iatrogenic trauma, intraperitonial bladder rupture

## Abstract

Iatrogenic intraperitoneal bladder injury is a known complication of pelvic surgery. While the standard key management of such cases is surgical intervention, conservative approaches can be utilized under specific circumstances. We are presenting a case of delayed diagnosis of iatrogenic intraperitoneal bladder injury following cesarean section, which was treated with urethral catheter bladder drainage.

## Introduction

Iatrogenic urinary tract injuries can have a significant effect on a patient’s morbidity. The two most common procedures associated with iatrogenic bladder injury are hysterectomies and cesarean sections. At one institution, iatrogenic bladder injuries occurred from obstetric and gynecological procedures in 65%, general surgical procedures in 22%, and non-endoscopic urological surgery in 13% of cases, a delay in diagnosis was more likely to occur during laparoscopic surgeries [1]. The standard management of intraperitoneal bladder injuries is surgical repair, however, there have been successful reports of trials of non-operative management of iatrogenic intraperitoneal bladder injuries avoiding surgical and anesthetic complications with remarkable results and outcomes [2]. We hereby report a case of intraperitoneal bladder injury following cesarean section that was unrecognized intraoperatively and was treated successfully with bladder drainage alone.

## Case presentation

**Figure 1:**
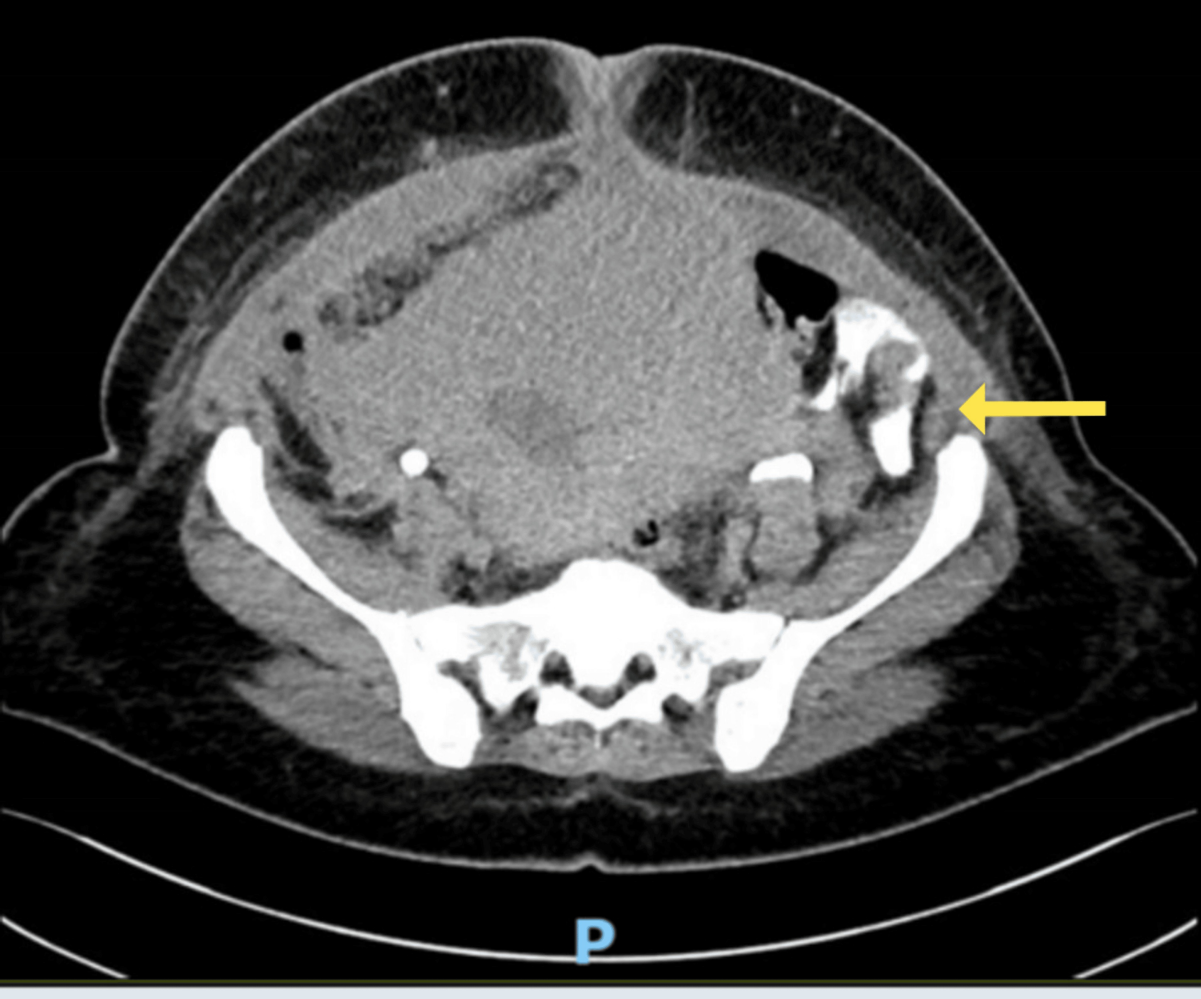
Axial view of the initial CT cystogram demonstrating extravasation of contrast that is spreading within the peritoneal cavity between the bowel loops (yellow arrow).

**Figure 2:**
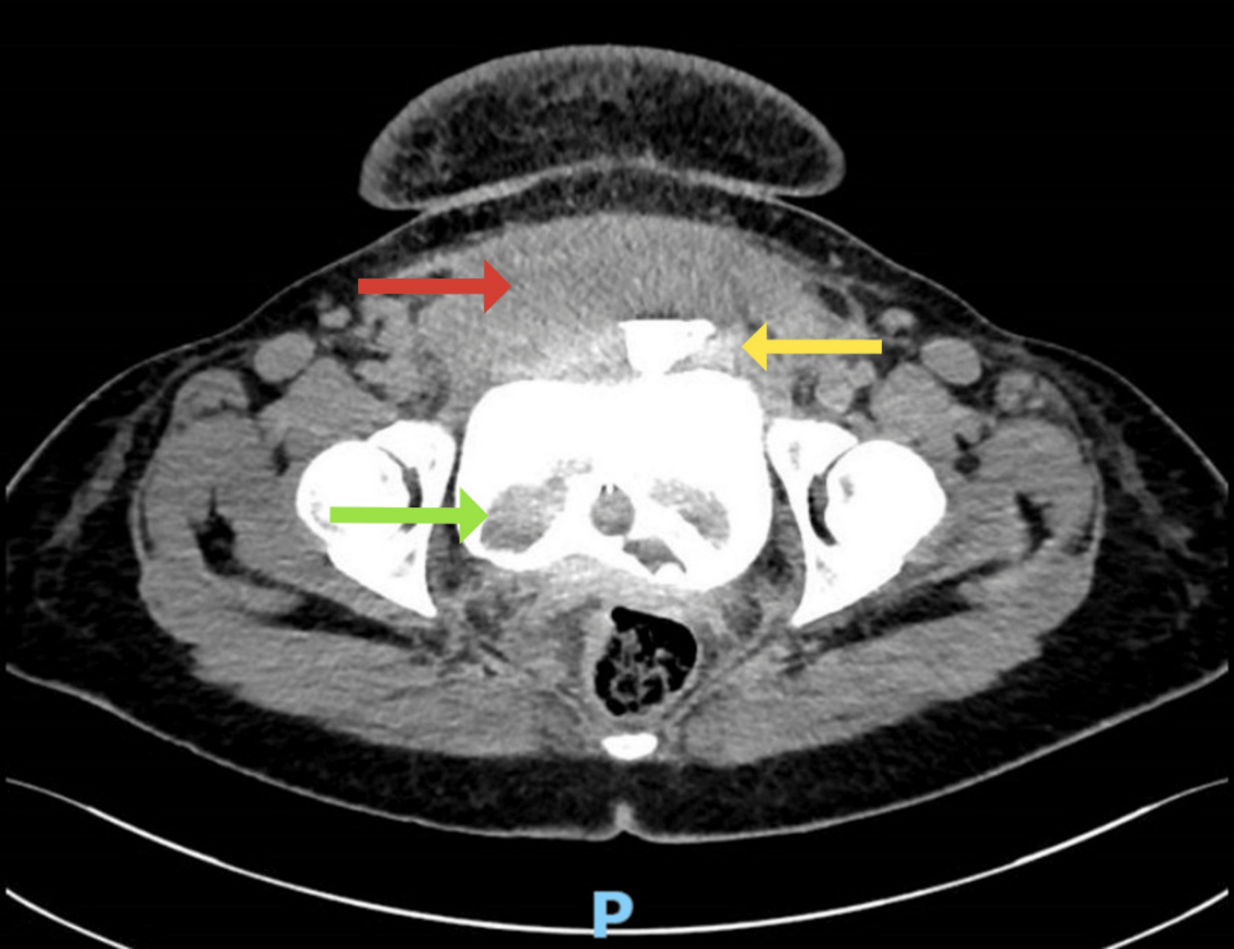
Axial view of the initial CT cystogram showing extravasation of contrast that is spreading above the urinary bladder (yellow arrow). There is significant pelvic fluid along the anterosuperior aspect of the bladder beneath the anterior abdominal wall measuring 111 x 88 x 38 mm with contrast leaking through this collection (red arrow). Urinary bladder shows catheter and blood clot within its lumen (green arrow).

**Figure 3:**
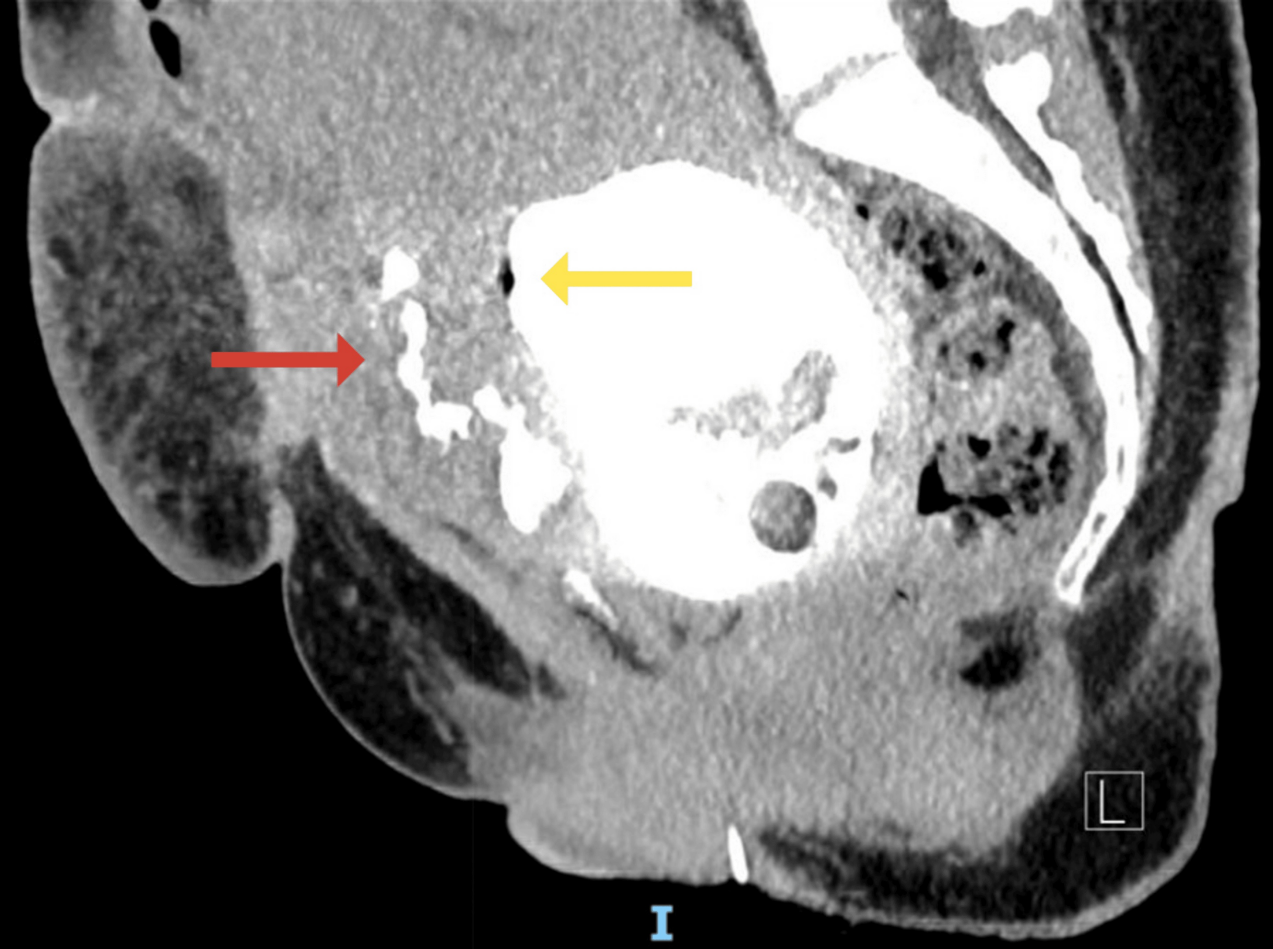
Sagittal view of the Initial CT cystogram demonstrating a small partial tear in the anterior wall of the urinary bladder measuring approximately 4 mm in diameter (yellow arrow) associated with extravasation of contrast. Redemonstration of an organized collection seen anterior to the bladder and lower uterine segment beneath the anterior abdominal wall with contrast leaking through this collection (red arrow).

A 37-year-old Saudi female patient with a past medical history of gestational diabetes mellitus (GDM) and a single previous cesarean section underwent an uneventful emergency cesarean section. On postoperative day one, the urethral catheter was removed and the patient voided freely without urinary symptoms or hematuria. She was discharged home on postoperative day three on good clinical status. On the same day of discharge, she presented to the emergency room (ER) primarily complaining of urge urinary incontinence and lower abdominal pain at the site of the cesarean section wound. She had no abdominal distention or fever. Complete blood count (CBC), renal function test (RFT), and serum electrolytes were all normal as a hemoglobin (HgB) level of 8.1 g/dL, hematocrit (Hct) of 25.8%, serum white blood cells (WBC) of 9.6 x10^3^ uL, creatinine levels of 1 mg/dL. Urine dipstick was positive for nitrite and leukocytes. Bedside ultrasound by the primary obstetrician was unremarkable. With a provisional diagnosis of presumed postpartum urinary tract infection (UTI), she was readmitted to the hospital under obstetrics and gynecology service. A urine culture was done, and she was empirically commenced on parenteral antibiotic (ceftriaxone). On routine nursing assessment, it was noticed that her underpad was almost always soaked with blood and she was presumed to have postpartum vaginal bleeding. On postoperative day six she developed acute urinary retention; a urethral catheter (two-way, 14Fr) was inserted with an immediate drainage of hematuria. A suspicion of urinary tract injury was raised, and urology was then consulted. On clinical evaluation, she was hemodynamically stable, a mild, non-tender distention at the lower abdomen below the cesarean wound was noted and the surgical wound itself was healthy with no signs of infection, dehiscence, or fluid leak. Stat CBC and RFT were normal with no significant change as the initial labs. Urgent computerized tomography urography (CTU) and computerized tomography (CT) cystogram were performed revealing a tear measuring 4 mm located at anteroinferior urinary bladder wall with intraperitoneal contrast extravasation associated with an organized pelvic collection anterior to the bladder and large vesical hematoma (Figures 1-3).

**Figure 4:**
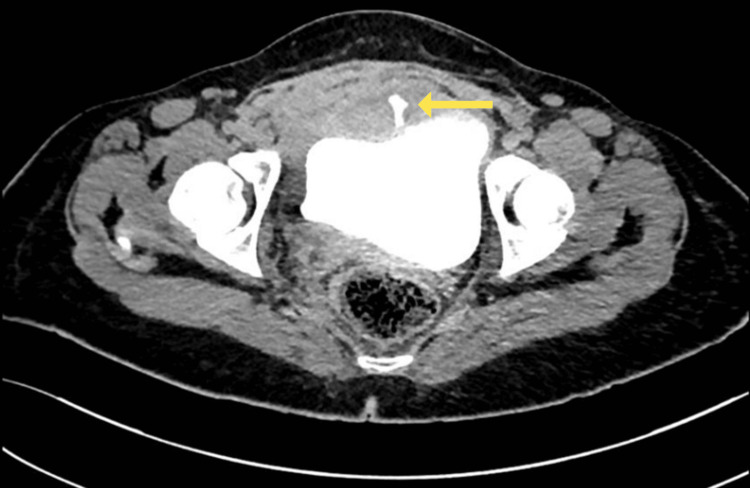
Axial view of the repeated CT cystogram after two weeks from the initial study showing contrast leakage along mid-line of the urinary bladder tear with smaller amount of leakage as compared to the initial scan (yellow arrow). Also noted a partial regression of the previously extraperitoneal collection anterior to the bladder.

Findings were discussed with the patient together with the different options of management and we agreed to go for a trial of conservative therapy. She was kept on a urethral catheter for a total of 14 days. In the first seven days, she had multiple attacks of catheter blockage and recurrent hematuria that were managed by changing the catheter to a larger size (two-way, 22Fr) and aspirating as many altered blood clots as possible without fluid irrigation. By the end of the first week, there were no more catheter blocks or hematuria. Urine continued to be clear, and she was started on an anti-cholinergic (solifenacin) as she was complaining of a catheter leak. CT cystogram repeated on the 14th day of urethral catheter placement showed improvement of intraperitoneal bladder extravasation in comparison to the initial study associated with regression of pelvic hematoma and resolution of vesical hematoma (Figure 4).

**Figure 5:**
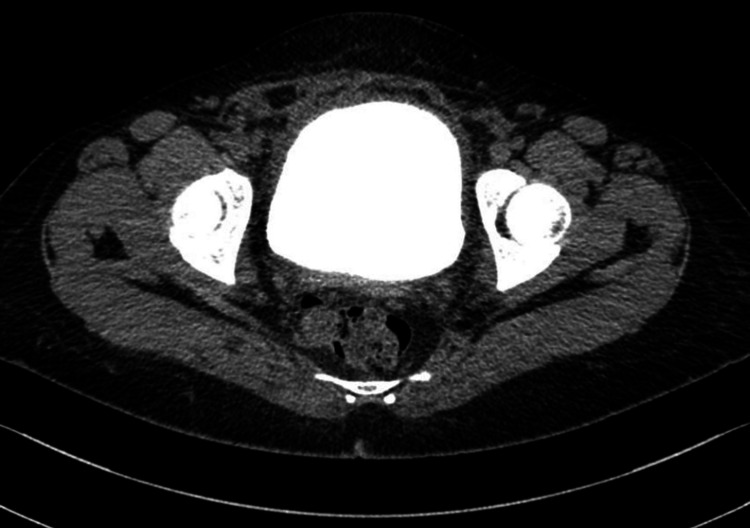
Axial view of the repeated CT cystogram after four weeks from the initial study showing a fully opacified, well-rounded urinary bladder with no evidence of contrast leakage and resolution of the anterior pelvic collection.

**Figure 6:**
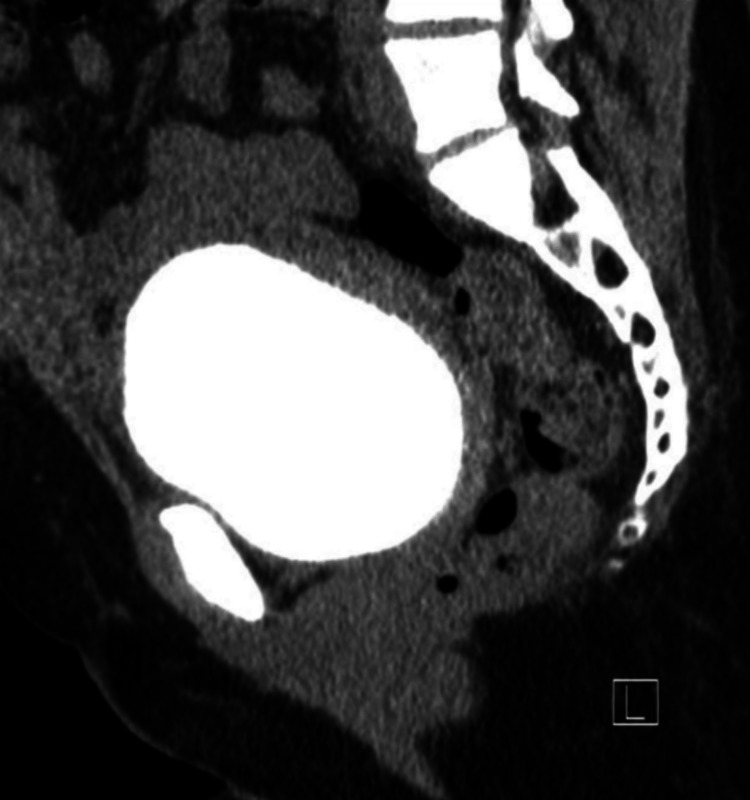
Sagittal view of the repeated CT cystogram after four weeks from the initial study showing a fully opacified, well-rounded urinary bladder with no evidence of contrast leakage and resolution of the anterior pelvic collection.

The decision was made to leave the urethral catheter for another trial of bladder drainage. Ensuring that the patient was clinically stable, she was discharged from the hospital with a catheter in place, an anti-cholinergic (solifenacin), and daily prophylactic oral antibiotic (nitrofurantoin) with a plan to repeat CT cystogram which was done two weeks later showing an intact bladder wall with no evidence of contrast extravasation (Figures 5, 6).

The voiding trial was successful after catheter removal. The patient was asymptomatic on her three-month appointment follow-up.

## Discussion

Generally, bladder injuries are categorized as non-iatrogenic penetrating or blunt injuries. Iatrogenic injuries are either caused by pelvic surgery or endourological procedures. Bladder injuries can be classified as contusion, extraperitoneal, intraperitoneal, or combined intra-extraperitoneal perforations. A grading system for bladder injuries also exists as grade 1: contusion, intramural hematoma or partial thickness laceration, grade 2: extraperitoneal bladder wall laceration <20 mm, grade 3: extraperitoneal bladder wall laceration >20 mm or intraperitoneal bladder wall laceration <20 mm, grade 4: intraperitoneal bladder wall laceration >20 mm, and grade 5: extra or intraperitoneal bladder wall laceration involving the trigone or bladder neck [3]. Most of the bladder injuries are caused by penetrating or blunt injuries. Iatrogenic bladder injuries usually have risk factors rather than causes that may include the number of previous cesarean sections and complicated pregnancies [4]. 

Intraoperatively they may be recognized by direct visualization of urine leakage from the bladder, bladder wall lacerations, bladder catheter protrusion, and blood or gas in the urine bag. Delayed presentation may appear as gross hematuria which is the most common and reliable sign, suprapubic or lower abdominal tenderness and bruises, abdominal muscles guarding and rigidity, abdominal distension due to urinary ascites, inability to void, or inadequate urine output. Unrecognized bladder injuries may manifest as uremia, azotemia, acidosis, fever, sepsis, ileus, or signs of peritonitis. Multiple radiological methods may aid in the diagnosis. Ultrasound alone is insufficient, although it can be used to visualize intraperitoneal fluid or an extraperitoneal fluid collection. Plain x-ray, cystogram, or CT cystography are the gold standard that shows contrast extravasation, or by cystoscopy.

Once diagnosed, management is based on multiple factors including time of recognition, type of injury, extent of injury, and clinical status of the patient. Two approaches are implicated, conservative or surgical, the former consists of clinical observation, bladder drainage, and prophylactic antibiotic, and it is the standard treatment for uncomplicated small extraperitoneal injuries. However, bladder neck involvement, bone fragments in the bladder wall, concomitant rectal or vaginal injury, or entrapment of the bladder wall necessitate surgical intervention to prevent complications such as fistula, abscess, and prolonged leak. 

Intraperitoneal injuries are usually managed by surgical repair because intraperitoneal urine extravasation can lead to peritonitis and intra-abdominal sepsis. However, there are multiple successful trials of conservative management for small intraperitoneal bladder injury by indwelling transurethral catheter alone, percutaneous peritoneal drain alone, or combined transurethral catheter and percutaneous peritoneal drain [5].

In our case, it was a delayed presentation of uncomplicated iatrogenic intraperitoneal bladder injury. Reviewing the operative report, there were severe tissue adhesions and multiple thick bands between the anterior abdominal wall and uterus managed by adhesiolysis. Overall, she had two possible reasons; one is from the surgery itself or from the initial forceful urethral catheter insertion compressing on a weak urinary bladder wall. Clinically she was hemodynamically stable, her blood work, electrolytes, and renal function were all within normal. At first, she was clinically improving after evacuation of vesical hematoma without irrigation. The addition of an anti-cholinergic is advised for optimal bladder stabilization. The abdominal collection was resolved spontaneously. A few similar reported cases in the literature exist [2,5,6] that were managed conservatively with excellent outcomes avoiding the morbidity of surgical interventions (Tables 1, 2).

**Table 1:** Relevant literature, initial management. CS; cesarean section, Fr; French, NCCT; non-contrasted computerized tomography, UB; urinary bladder, US; ultrasound, TVH; total vaginal hysterectomy, CH; cesarean hysterectomy, CCT; contrasted computerized tomography, TAH; total abdominal hysterectomy.

Literature	Presentation	Management
Albukhari et al. [2]	A 35-year-old female developed abdominal distention and gross hematuria six hours post lower abdominal CS	-Bladder drainage via urethral catheter (16Fr) -Intraperitoneal fluid drainage via drain placement (6.5Fr) -NCCT showed large amount of intraperitoneal fluid collection -CT cystogram showed defect at the dome of the UB with intraperitoneal contrast extravasation
Aghaways et al. [5]	A 35-year-old female developed abdominal distention, gross hematuria, and difficulty voiding 11 days post elective CS	-Bladder drainage via urethral catheter -Intraperitoneal fluid drainage via drain placement (12Fr) -US showed marked abdominal ascites -Cystoscopy showed defect at posterior UB wall
Alperin et al. [6]	Case #1: A 45-year-old female developed abdominal discomfort and urine retention one day post TVH	-Bladder drainage via urethral catheter (16Fr) -NCCT showed perivesical fluid collection -Cystoscopy showed defect at posterior UB wall measuring 15 mm -Gravity cystography confirmed intraperitoneal bladder injury
	Case #2: A 28-year-old female developed abdominal distention and oliguria one day post CH	-Bladder drainage via urethral catheter (16Fr) -CCT showed intraperitoneal extravasation -Cystoscopy showed defect at posterior UB wall measuring 15 mm -Gravity cystography confirmed intraperitoneal bladder injury
	Case #3: A 45-year-old female developed oliguria one day post TAH	-Bladder drainage via urethral catheter (16Fr) -NCCT showed free pelvic and perihepatic fluid collection -Cystoscopy showed defect at UB dome measuring 20 mm -Gravity cystography confirmed intraperitoneal bladder injury

**Table 2:** Relevant literature, follow-up. CTU; computerized tomography urography, VCUG; voiding cystourethrogram.

Literature	Follow-up
Albukhari et al. [2]	Discharged with urethral catheter and abdominal drain in place. Follow-up CTU after two weeks showed complete resolution with no contrast extravasation.
Aghaways et al. [5]	Discharged with urethral catheter in place. Follow-up VCUG after two weeks showed no contrast leak or extravasation.
Alperin et al. [6]	Case #1: Discharged with urethral catheter in place and prophylactic oral antibiotic (nitrofurantoin). Follow-up VCUG after two weeks showed no contrast leak or extravasation.
	Case #2: Urethral catheter left in place. Follow-up VCUG after 20 days showed extravasation during filling phase, repeated VCUG after 14 days showed lesser degree of extravasation during voiding phase, repeated VCUG after another 14 days showed no contrast leak or extravasation.
	Case #3: Discharged with urethral catheter in place and prophylactic oral antibiotic (nitrofurantoin). Follow-up VCUG after 17 days showed no contrast leak or extravasation.

## Conclusions

Management of intraperitoneal bladder rupture is primarily surgical repair, however, grade 3 intraperitoneal injuries can be either surgical or conservative depending on their severity and timeline of presentation. Delayed uncomplicated cases in terms of cold cut injuries, absence of surrounding organ injuries, or gross peritoneal contamination can be successfully managed with a conservative approach initially followed by conversion to surgical therapy if required. Hence, every case should be individualized and vigilantly assessed to elect such an approach.
